# An iterative, user-centered design process to guide the development of a virtual reality (VR) experience that supports motivational interviewing for young adult drinking

**DOI:** 10.21203/rs.3.rs-10658559/v1

**Published:** 2026-08-14

**Authors:** Tyler Blake Wray, Erik M.S. Ocean, Amy C. Traylor, Molly Magill, Nancy P. Barnett, Christopher W. Kahler, Ruben G. Martinez

**Affiliations:** Brown University; Brown University; University of Alabama System; Brown University; Brown University; Brown University; Brown University

**Keywords:** Virtual reality, alcohol use, motivational interviewing, young adults

## Abstract

**Background:**

Brief motivational interventions (BMIs) reduce hazardous drinking among young adults, but their effects are modest, prompting interest in enhancements. BMIs may work partly by developing discrepancy between clients' current behavior and important life priorities or values. Virtual reality (VR) has been used to complement addiction treatment and facilitate transformative experiences. We developed *Fresh Start*, a VR experience designed to make this discrepancy tangible within a BMI, and evaluated it through iterative user research.

**Methods:**

We used a mixed-methods, build-test-refine design across three cycles with 32 hazardous-drinking young adults (ages 18–35). In each cycle, participants completed a mock BMI and reviewed a progressively more complete VR prototype, from 2D still images (Cycle 1) to a “grey box” build (Cycle 2), and finally a fully-functional version embedded in a complete BMI session (Cycle 3). Participants thought aloud while using the prototype, then completed a semi-structured interview and an online survey rating acceptability, feasibility, appropriateness, usability, tolerability, and engagement.

**Results:**

Participants found *Fresh Start* acceptable, feasible, and appropriate, with ratings near the top of each scale (*M*s = 4.2–4.3 on a 1–5 scale) and higher ratings as prototype sophistication increased. Usability was good in both functional builds (Cycle 2 System Usability Scale *M* = 84.6, *SD* = 8.5; Cycle 3 *M* = 75.0, *SD* = 9.0). Functional builds were safe and well tolerated, with mild, transient symptoms, low simulator sickness scores, and no serious adverse events. By Cycle 3, when counselors asked what Fresh Start made them think about, nearly all participants reflected on the intended discrepancy.

**Conclusions:**

*Fresh Start* is an acceptable, feasible, and safe way to incorporate VR into BMIs for young adults, and it reliably prompts users to reflect on the discrepancy it was designed to highlight. Future research will conduct a fully powered randomized controlled trial testing its effect on alcohol-related outcomes among mandated college students.

## Introduction

Despite recent reductions in drinking among young adults, alcohol remains the leading risk factor for premature death in this age group (Esser et al., 2024; Griswold et al., 2018) and heavy drinking is still prevalent, with 37% of 18–25 year olds reporting at least one binge drinking episode (> 5 drinks for men or > 4 drinks for women on a single occasion) in the past month (Substance Abuse and Mental Health Services Administration, 2018). Binge drinking in young adulthood also increases risk for chronic health conditions, such as obesity and high blood pressure, later in life (Oesterle et al., 2004). Research exploring ways to reduce hazardous drinking among young adults continues to be an important public health priority.

Interventions based on the principles of Motivational Interviewing (MI) are effective in helping recipients reduce their alcohol use and related problems (Burke et al., 2003; Imel et al., 2008). MI-based approaches to counseling are also widely used among addiction counselors in community settings (Ball et al., 2002; T. B. Wray, 2022). Brief motivational interventions (BMIs), which are single-session, directive counseling sessions that incorporate MI techniques, have been shown to reduce alcohol use and problems in a range of populations, including young adults (Burke et al., 2003; Carey et al., 2007; Ray et al., 2014; Tanner-Smith & Lipsey, 2015). Due to their single-session format and non-confrontational approach, these interventions are well-suited for delivery in settings that have contact with people who drink hazardously but are not necessarily seeking treatment, like emergency departments and college counseling centers (Bray et al., 2017; Finnell, 2013; Gaume, Magill, et al., 2014; Pringle et al., 2012). However, recent meta-analyses, including one using individual participant data, have suggested that the effects of BMIs may be modest overall (*d*=−0.10 to −0.15 relative to assessment only and alternative interventions) prompting interest in ways to augment these effects (Foxcroft et al., 2016).

Although it is still unclear exactly how MI or MI-inspired interventions like BMIs help patients reduce their drinking, one potential mechanism is through eliciting and accentuating the degree of discrepancy patients feel between their current behavior and broader life priorities and values (Gaume, McCambridge, et al., 2014; McNally et al., 2005). Discrepancy refers to a “deepened, conscious incongruity between one’s actual and ideal drinking patterns” (McNally et al., 2005, p. 80). Developing discrepancy was formerly one of the four key principles of MI (W. R. Miller et al., 2012). There is some evidence that patients’ post-session sense of discrepancy is most consistently associated with outcomes (Apodaca & Longabaugh, 2009). A variety of techniques in MI and BMIs are intended to help develop discrepancy. A key part of providing personalized normative feedback about drinking, a centerpiece of many BMIs, involves highlighting the inconsistency between patients’ own drinking and their perceived norms of others’ drinking, which can increase patients’ sense of self-other discrepancy (Borsari & Carey, 2003). Likewise, asking open-ended questions that encourage patients to reflect on how current behavior aligns with personal values or goals can increase their sense of actual-ideal discrepancy (McNally et al., 2005; W. R. Miller et al., 2012; W. R. Miller & Rose, 2009), as can double-sided reflections that bring discrepant motivations into focus.

## Virtual Reality as a counseling tool

Virtual reality (VR) refers to computer-generated environments that create an immersive illusion of reality (Slater & Wilbur, 1997). VR is unique among technologies in producing a sense of *presence* in users, a convincing sense of feeling as though one is actually *in* a virtual environment (Penn & Hout, 2018; Riva et al., 2003). It creates this sense of presence by delivering synchronized multisensory input (visual, auditory, and spatial) that mirrors how humans naturally perceive their environment. For example, visual scenes update in real time with head movement, and sounds shift in volume and direction based on a user's position.

This sense of presence is what makes VR a potentially powerful tool for counseling and behavior change. For example, one application of VR helps treat anxiety disorders by facilitating exposures (Carl et al., 2019; Deng et al., 2019; Morina et al., 2015), which involve confronting a feared situation so that patients can learn that these situations are not dangerous or intolerable (Craske et al., 2008). Meta-analyses have shown VR-assisted exposures are as effective as *in vivo* exposures (Carl et al., 2019; Deng et al., 2019), and that they generalize to real-world contexts (Morina et al., 2015; Opriş et al., 2012), while potentially being easier for clinicians to implement (Wray et al., 2022). Another application of VR involves simulation-based learning, which helps patients practice skills in environments that are similar to those they will need to use them in (Grabowski & Jankowski, 2015; Seymour et al., 2002). A third potential application of VR involves using immersive experiences to cultivate emotional states that could prompt deeper reflection and motivate change. Research suggests that VR can reliably elicit feelings of awe and wonder, and that these states are associated with prosocial attitudes (Quesnel et al., 2018; Quesnel & Riecke, 2017; Stepanova et al., 2018). VR could also foster a sense of curiosity and openness that helps patients be more receptive to new perspectives (Herrera et al., 2018; Quesnel & Riecke, 2017; Stepanova et al., 2018).

Applications of VR to addiction treatment have so far been limited. Although a few studies have explored whether VR can help facilitate mindfulness or coping skills (Faraj et al., 2021; Garland et al., 2024; Thaysen-Petersen et al., 2024; Van Doren et al., 2024), the vast majority have used VR to attempt to reduce “cue reactivity,” or a conditioned craving responses related to drug cues (Ghiţă et al., 2025; Huang et al., 2025; Zhang et al., 2023). Pilot studies of these experiences have shown some promise (Garland et al., 2024; Huang et al., 2025; Zhang et al., 2023), but no fully powered trials of their effects on meaningful, long-term outcomes have been published.

### Study Aims

The aim of the present study was to design, build, and refine prototype and refine *Fresh Start*, a VR experience designed for use within BMI for alcohol use sessions to help counselors elicit client change talk (i.e., statements about making a change in behavior) by making their sense of discrepancy more tangible (Wray, et al., In review). In the experience, users are asked to imagine they are moving to a new place and are walking through a virtual apartment collecting objects (called tokens) that represent life priorities that may be important to them, such as education, career, travel, romantic relationships, or family. When they bring these objects to the waiting moving truck, they find it is already largely occupied by a representation of their alcohol use, which requires participants to physically resolve this conflict by selecting items to bring or leave behind. This design feature thus requires participants to consider the trade-off (discrepancy) between their drinking and various life priorities. We designed the experience to be consistent with the tone of MI as much as possible, meaning it emphasizes collaboration and attempts to draw out users’ own values and reasons for change rather than imposing them. The overall goal of *Fresh Start* is to give counselors a concrete tool for helping clients explore and resolve ambivalence by illustrating, rather than simply discussing, the conflict between their drinking and their priorities. See Wray et al. (In review) for a more detailed description of the experience.

To ensure *Fresh Start* was grounded in the perspectives of its intended users before evaluating its impact on outcomes, we engaged 32 hazardous-drinking young adults to develop the experience through three iterative cycles of user research. In each cycle, the experience was progressively more complete, starting with renderings of key concept screens in Cycle 1 and ending with a complete, fully-functional version in Cycle 3. Each cycle used mixed methods to evaluate participants’ interest in the experience (acceptability), the feasibility of using it within a BMI session; its appropriateness for young adults; usability, tolerability, participants’ engagement with VR generally and with the premise of this experience; and their perceptions of how consistent the experience was with the “tone” of MI. This manuscript describes the results of these user research cycles.

## Methods

### Study Design

This study employed a mixed-methods, build-test-refine design to iteratively develop the *Fresh Start* VR experience with feedback from users (see Fig. 1). Each cycle used a distinct set of participants. In each cycle, participants attended an in-person interview session in which they first engaged in a mock BMI to provide them with adequate context for feedback. Then, each participant reviewed that cycle’s prototype in the VR headset (Oculus Quest 3, Menlo Park, CA), and were asked to “think aloud” when interacting with the experience. After participants finished reviewing the prototype, staff conducted a semi-structured interview and participants completed a short survey. After each cycle, feedback was compiled and used to generate a prioritized list of design requirements for the subsequent cycle.

## Participants

Across all three cycles, eligible participants were: (1) young adults (ages 18–35), (2) who reported drinking “hazardously” in the past month, defined by NIAAA guidelines as consuming more than 14 drinks per week (men) or 7 drinks per week (women), or more than 5 drinks on a single occasion (men) or 4 drinks (women) (National Institute on Alcohol Abuse and Alcoholism, 2005), and (3) were fluent in English. Participants were excluded if they: (1) reported currently receiving counseling or medication for a substance use disorder, (2) reported injection drug use in the past three months, (3) reported having a serious mental illness (e.g., schizophrenia, severe bipolar), (4) reported having experienced symptoms of alcohol withdrawal when cutting down or stopping their drinking in the past, (5) had a history of epilepsy or other seizure disorder, (6) had ever been told by a medical professional that they should not use devices with bright lights or a lot of visual motion, and (7) had a score of < 6 on the Visually Induced Motion Sickness Susceptibility Questionnaire (Keshavarz et al., 2021). Participants were recruited primarily via social media advertisements.

## Procedures

Interested participants completed an eligibility screening survey online. Those eligible were provided with further information about the study and asked to indicate their informed consent online. Those who consented then provided their contact information and completed a baseline survey. Afterward, research staff contacted participants to schedule their in-person session, which lasted approximately 90 minutes. During the sessions, the interviewer first engaged the participant in a 60-minute BMI session (see BMI section below for details). In Cycles 1 and 2, interviewers completed the BMI topics (building rapport, personalized normative feedback, pros and cons, assess motivation) before introducing the idea of VR and asking participants if they were interested in trying it (“I actually have a virtual reality experience that might help you reflect on this issue a bit more. Would you like to try it?”). Interviewers mirrored the VR headset to a computer so they could monitor what the participant viewed, handed participants the headset, and provided brief (< 3 min.) instructions about how to fit the headsets on their head, adjust pupillary distance, and use the controllers. Then, the interviewer instructed participants to start the *Fresh Start* app. The prototype reviewed in each cycle represented a progressively more complete version of the experience. Cycle 1 used five 2D still images that depicted the basic scenes of the experience that were displayed within the VR headset. Interviewers then narrated the premise of the experience and what participants would be asked to do in each scene. Cycle 2 used a functional “grey box” 3D prototype. A “gray box” prototype is a fully-functional VR environment that allows users to navigate normally and interact with tokens, but uses basic, gray 3D shapes in the environment to test spatial layout and movement before adding final artwork and textures. Cycle 3 used a fully functional version with all artwork included. We had originally planned for up to five cycles of user research to support development but found that three provided sufficient data for making the design decisions needed to generate the next prototype. In Cycles 2 and 3, once participants started the *Fresh Start* app, they were allowed to play the experience normally with no outside instructions provided by the interviewer. In Cycle 3, terviewers also completed a full mock BMI session, including topics both before and after the VR. Participants were informed that the purpose of the interview was to elicit feedback about new approaches to counseling, and to facilitate authentic feedback, we would conduct the full counseling session as planned, but that they were welcome to invent details about themselves or respond authentically. We approached it this way to attempt to provide a more accurate picture of the “final” feasibility and acceptability of the VR-enhanced BMI, but also to help the research team assess how VR might be best introduced and processed afterward in the context of a more realistic interview and the timing of session elements. Across all sessions, participants were asked to “think aloud” as they reviewed the prototype, which is an approach to design interviews and usability testing in which participants verbalize their thoughts, reactions, and confusion as they interact with a prototype (Van Den Haak et al., 2003). This allows researchers to capture reactions other methods might miss. After reviewing the VR (Cycles 1 & 2) or completing the full session (Cycle 3), participants then completed a semi-structured interview. Questions inquired about participants' overall impressions, sense of presence and immersion, usability, and whether the experience prompted reflection on their personal goals, alcohol use, and the potential conflicts between the two. Participants also completed an online survey after the session on their own at home that assessed acceptability, feasibility, and appropriateness of the experience, as well as usability, presence, flow, and simulator sickness. Participants were compensated $100 for completing all steps of the study. All procedures were approved by the Brown University Institutional Review Board.

### Brief Motivational Intervention (BMI).

The BMI we used in this study was based on Barnett et al. (2019). The session opened with counselors setting a collaborative, non-judgmental tone, followed by a review of the participant’s drinking and personalized normative feedback comparing their frequency, quantity, binge frequency, and estimated monthly drinks against national college-student norms. Next, counselors elicited reactions and guided a discussion about the pros and cons of their drinking level. The counselor then introduced VR, assessed participant interest, oriented them to the headset, and allowed them to use the experience without instructions. Afterward, counselors used MI techniques to elicit change talk and administered importance and confidence in change ruler items. Participants interested in change then developed a plan with goals, strategies, anticipated challenges, and potential sources of support, while those not yet interested engage in a “planting a seed” discussion about what would need to happen for them to consider change. Session lasting roughly 60 minutes. Figure 1 depicts the key topics covered in these sessions and how we integrated VR into them.

#### VR experience

The *Fresh Start* experience was built for the Oculus Quest line of VR head-mounted display systems using the Unity Engine. The experience begins with a brief orientation to VR and its controls, and a short survey about users’ typical drinking patterns and beverage preferences, which is used to personalize a key scene later in the experience. Users are then introduced to the plot, which involves asking them to imagine that they are moving to a new place to start a new chapter of their lives. Next, they are asked to walk through a virtual apartment and select tokens that represent different life priorities (e.g., a diploma for education, a briefcase for career) and rate the importance of each one to them. A rating of “extremely important” packs 3 boxes representing the object, “very important” packs two boxes, “not very important” packs 1 box, and opting out of the object doesn’t pack any boxes. A written description and matching narration describes each token as users pick it up. After they finish, participants are guided through a doorway and outside where there is a moving truck in which part of the cargo space is already occupied by alcohol matching their previously selected beverage preference (e.g., images of cases of beer). The amount of “space” the alcohol takes up is scaled to match the participant’s reported drinking level. Since space is limited, most participants will not be able to fit in all the things they brought from the apartment, so must decide to either remove some alcohol or leave some important life priorities behind. After they have completed their “move,” a summary screen shows what they packed and what they left behind. The experience was designed to last approximately 12–15 minutes. Afterward, in the second part of the BMI session, counselors asked participants to describe what the experience prompted them to think about, and applied MI techniques to elicit change talk.

## Measures

**Alcohol use** was assessed using the AUDIT-C (Bush et al., 1998), a 3-item abbreviated version of the Alcohol Use Disorders Identification Test (AUDIT) with items measuring frequency of drinking, typical quantity, and frequency of heavy episodic drinking. Each item is scored from 0 to 4. Participants who reported drinking ≥ 14 drinks per week (men) or ≥ 7 drinks per week (women), or more than 5 drinks on a single occasion (men) or 4 drinks (women), were classified as “drinking hazardously” (National Institute on Alcohol Abuse and Alcoholism, 2005). The AUDIT-C has demonstrated good sensitivity and specificity for detecting hazardous drinking in both clinical and community samples (Bradley et al., 2003; Bush et al., 1998).

**Alcohol craving** was assessed using a single-item visual analogue scale on which participants rated their urge to drink from 0 = *no urge at all* to 100 = *strongest urge imaginable*. Single-item visual analogue scales of craving have been widely used in substance use research (Drobes & Thomas, 1999) and have demonstrated good convergent validity with multi-item craving measures, sensitivity to experimental manipulation, and predictive validity for subsequent drinking (Sayette et al., 2000).

**Actual-ideal discrepancy** related to alcohol use was assessed using a single-item measure adapted from McNally et al. (2007) in which participants rated the following item on a scale of 0 = *I’m now at my ideal* to 10 = *I’m very far from my ideal*: “How close or far do you think your current drinking patterns are from your personal ‘ideal,’ given who you are as a person, your life circumstances, your interests, and your values?”

**Acceptability, feasibility, and appropriateness** were assessed using the Acceptability of Intervention Measure (AIM), Feasibility of Intervention Measure (FIM), and Intervention Appropriateness Measure (IAM) (Weiner et al., 2017). Participants rated the extent to which they found the *Fresh Start* experience to be acceptable, feasible, and appropriate for use in a counseling context. Each scale had 4 items rated on a 5-point scale (1 = *completely disagree* to 5 = *completely agree*). Example items include “*Fresh Start* seems appealing to me” (AIM) and “*Fresh Start* seems implementable” (FIM). Previous studies have shown that the AIM, FIM, and IAM have strong internal consistency and construct validity across a range of health intervention contexts (Weiner et al., 2017)(AIM α = 0.85, FIM α = 0.71, IAM α = 0.94).

**Usability** was assessed using the System Usability Scale (SUS) (Brooke, 1996; Sauro, 2011), a 10-item scale on which participants rate their agreement with alternating positive and negative usability statements on a 5-point scale (1 = *strongly disagree* to 5 = *strongly agree*). Scores are converted to a composite ranging from 0 to 100, with scores of 68 or above considered “above the industry average” (Bangor et al., 2008). Example items include “I thought the system was easy to use” and “I needed to learn a lot of things before I could get going with this system.” The SUS is one of the most widely used measures of perceived usability and has demonstrated acceptable reliability across a range of technology contexts (Lewis & Sauro, 2009)(α = 0.77).

**Presence**, or participants’ subjective sense of being in a virtual environment, was assessed using the iGroup Presence Questionnaire (IPQ) (Schubert et al., 2001). The IPQ is a 14-item scale (range: 14–70), rated on a 1 *(fully disagree*/*not at all*) to 5 (*fully agree/very much*) scale, that includes example items such as “somehow, I felt that the virtual world surrounded me,” and “I had a sense of ‘being there.’” The IPQ has shown good psychometric properties in VR research contexts (Tran et al., 2024)(α = 0.92).

**Flow**, or a state of task-related absorption and enjoyment, was assessed using the Flow Short Scale (Rheinberg et al., 2003). This scale consists of 10 items, each rated on a 1 = *not at all* to 7 = *very much* scale (range: 10–70). Example items include: “I did not notice time passing,” and “I was completely lost in thought.” The Flow Short Scale has demonstrated good internal consistency and predictive validity across multiple types of tasks (Engeser & Rheinberg, 2008)(α = 0.76).

### Tolerability.

We used the Simulator Sickness Questionnaire (SSQ) to assess the presence and severity of symptoms of motion sickness that can sometimes accompany use of simulators and VR (Kennedy et al., 1993). The SSQ includes 16 items (e.g., nausea, dizziness, blurred vision) rated on a 4-point scale (0 = *none* to 3 = *severe*). The SSQ has been widely used in VR research (Mazloumi Gavgani et al., 2018; Rebenitsch & Owen, 2016)(α = 0.86). We also collected the Patient-Reported Outcomes version of the Common Terminology Criteria for Adverse Events (PRO-CTCAE), a checklist of 38 potential symptoms. For each symptom selected, participants were asked to report their severity on a 0 = *none* to 4 = *very severe* scale, as well as the extent to which each symptom interfered with usual or daily activities.

### Consistency with MI tone.

A 13-item custom scale we have used in previous work (T. B. Wray et al., 2021) assessed whether participants perceived that the VR experience was consistent with MI’s tone, including its support of users’ autonomy, empathy, and evocation. Example items include “the experience showed respect for me” and “the experience was supportive of my ability to decide for myself.” Participants rated items on a 0 = *strongly disagree* to 4 = *strongly agree* scale. Finally, we also asked participants in Cycle 3, who were exposed to all topics covered in the planned BMI session, to pick and rank topics that (1) were most memorable to them, and (2) changed their perspective on their drinking the most. Example topics included: “comparing your drinking with other young adults,” “reflecting on the pros and cons of your drinking,” and “thinking about your life priorities and how drinking fits (the VR experience).” Since scores from these items reflected both whether participants picked each topic and its rank across two items, we scored each topic using a positional rank-sum approach (Emerson, 2013). In this approach, each topic earned points based on where participants ranked it (6 points for 1st place down to 1 point for 6th, 0 if not picked), and points were averaged across participants. Higher scores mean that more participants picked the topic and ranked it highly.

#### Data analysis

Qualitative data from the semi-structured interviews were analyzed using a directed content approach (Hsieh & Shannon, 2005). The first author (TBW) reviewed each interview transcript using a set of a priori questions derived from the study's design goals for that cycle as an organizing framework. A second author (CP) then independently read the same transcripts to identify additional evidence elaborating or qualifying the primary findings. Quantitative data were analyzed using basic descriptive statistics in Stata 17 SE (College Station, TX). Chi-square tests (for categorical data) and *t*-tests (for continuous data) were used to compare across groups or cycles.

## Results

### Participant characteristics

A total of 32 young adults completed study sessions across the three cycles (Cycle 1: *N* = 8, Cycle 2: *N* = 9, Cycle 3: *N* = 15). Overall, participants had a mean age of 22.8 years (SD = 3.3) and were predominantly female (58%), White (81%) and non-Hispanic (82%). Nearly half of participants were full-time students (49%) with a third employed full-time (33%). Twelve percent of participants had AUDIT scores (≥ 16) that suggested high-risk drinking. Most participants had at least some prior VR experience (73%), though almost none had used it within the past month. Full demographic and baseline characteristics by cycle are presented in Table 1.

### Acceptability

All participants across all cycles (100%) agreed to try VR when asked in the context of a BMI, despite not being explicitly reminded that VR might be involved in their participation. In Cycle 1 post-VR interviews, several participants spontaneously noted that using VR might be more engaging than a standard counseling conversation: “*I think like doing a VR experience when you are forced to come for counseling is probably more fun than sitting and talking to someone*” (Participant #1, Cycle 1), and “*it feels more engaging, like in the head. You're listening to yourself a lot more. In that sense, I think it helps you get into your head a little better*” (Participant #2, Cycle 1). However, this perspective was not universal. One participant expressed a preference for direct conversation with a counselor. In Cycle 3, where participants experienced the fully functional build embedded in a complete BMI session, acceptability reactions were uniformly positive: “*It’s thought-provoking and it’s fun*” (Participant #29, Cycle 3), and “*I think the VR would be a really great experience for a lot of people*” (Participant #24, Cycle 3). Quantitative AIM scores reflected this pattern, with means above 3.9 out of 5 across all three cycles and a trend toward higher ratings in Cycles 2 and 3 relative to Cycle 1, though differences across cycles were not statistically significant (Cycle 1: *M* = 3.97, *SD* = 0.85; Cycle 2: *M* = 4.50, *SD* = 0.47; Cycle 3: *M* = 4.27, *SD* = 0.44; *p*=.176; see Table 2).

### Feasibility

Probably the most useful feedback about feasibility can be drawn from Cycle 3, when the experience was integrated into a full BMI session. Participants in this cycle consistently described the VR component as fitting smoothly into the flow of the session. One noted that, “*[it] didnt’ feel abrupt or anything, it kind of like flowed in*” (Participant #25, Cycle 3), while another reflected that, “*a lot of the components of the conversation also kind of subtly reached into the things that the VR touched on. The second I had the goggles on, I kind of forgot you guys were here because you're so immersed in it*” (Participant #31, Cycle 3). Quantitative feasibility ratings on the FIM reflected this, with high scores across all three cycles (Cycle 1: *M* = 4.59, *SD* = 0.38; Cycle 2: *M* = 4.50, *SD* = 0.56; Cycle 3: *M* = 4.20, *SD* = 0.43). Differences across cycles were not statistically significant (*p*=.110). See Table 2.

### Appropriateness

Participants also generally felt the VR experience was appropriate for use in a counseling setting and that it complemented rather than disrupted the session: “*It was cool, and cool that it like tied into kind of what we were talking about*” (Participant #26). Another mentioned that “*As soon as you saw [the tokens] in the room, it was like, oh yeah, I do think about these things, and they are relevant to me and my life currently*” (Participant #13, Cycle 2). However, participants’ ratings of appropriateness were lower in Cycle 1 (*M* = 3.66, *SD* = 1.18) than in Cycles 2 and 3 (Cycle 2: *M* = 4.33, *SD* = 0.54; Cycle 3: *M* = 4.22, *SD* = 0.44), although these differences were not statistically significant (*p*=.12). See Table 2.

### Usability

Since participants were interacting with static, 2D images in Cycle 1, the primary usability concern was whether the concepts for instructions and prompts were sufficiently clear enough to help participants understand the premise. Several initially treated token selection as though they were choosing literal objects rather than tokens that were symbolic of their priorities. This prompted us to make several changes to the instructions and token interaction flows before Cycle 2. In Cycle 2, users evaluated a “gray box” prototype, so navigation and interaction mechanics became the central usability focus. In this cycle, participants identified problems exiting the apartment, difficulty with token selection card orientation, inability to revisit previously considered tokens, and accidental exits from the apartment when interacting with tokens near the door. These issues were addressed before Cycle 3. In Cycle 3, residual usability issues were more minor, including head rotation constraints and unclear instructions about the ability to unpack boxes already loaded in the truck (“*I saw the boxes and I was like, 'Wait, I can unpack this?'“* [Participant #39]). Quantitatively, SUS scores exceeded the commonly used threshold of 68 in both Cycles 2 and 3, with means suggesting good usability (Cycle 2: *M* = 84.57, *SD* = 8.46, Cycle 3: *M* = 75.00, *SD* = 9.03). Total scores were significantly higher in Cycle 2 compared to Cycle 3 (*p*=.040; Table 2, Fig. 1).

### Tolerability

No participants discontinued VR use before completing the demo or the experience in any cycle, and no serious adverse events occurred in any cycle. In Cycle 1, where participants viewed static 2D concept images, only one participant (12.5%) reported any adverse symptom, a single episode of mild headache with no functional impairment, and SSQ scores were very low (*M* = 0.13, *SD* = 0.13 on a 0–2 scale). In Cycle 2, symptom endorsement increased: 5 of 9 participants (55%) reported at least one symptom on the PRO-CTCAE, most commonly dizziness (33%), headache (33%), and blurred vision (11%). All symptoms were rated as mild to moderate in severity, and no participant reported any functional impairment. SSQ scores remained low (*M* = 0.17, *SD* = 0.24). In Cycle 3, 7 of 15 participants (47%) of participants reported at least one symptom. Nausea was the most commonly endorsed symptom (31%), followed by dizziness (25%) and headache (19%). One participant directly linked their nausea to navigation in the truck scene: “*I felt a little bit nauseous in the truck. It was a little bit tricky going up the runway. That might have been what made me a little bit nauseous*” (Participant #26, Cycle 3). Severity ratings remained mild to moderate, and reported interference with daily activities was minimal across all endorsed symptoms. SSQ scores were higher but still low in Cycle 3 (M = 0.31, SD = 0.41). Differences in SSQ scores across cycles were not statistically significant (*p*=.354; Table 4, Fig. 6). Full adverse event data by symptom, cycle, severity, and interference are presented in Supplementary Table 1. Craving did not change significantly within sessions in any cycle (e.g., Cycle 3: pre *M* = 14.6, *SD* = 23.1 vs. post *M* = 18.1, *SD* = 28.9, *p*=.657; see Table 3, Fig. 2).

### Engagement with the VR experience

#### Presence and flow

Presence and flow scores increased from Cycle 1 to Cycles 2 and 3, consistent with the progressive increase in prototype sophistication across cycles (Table 3, Fig. 3). Presence scores were lowest in Cycle 1 (*M* = 2.89, *SD* = 0.71), where participants viewed 2D still images, and higher in Cycles 2 (*M* = 3.45, *SD* = 0.19) and 3 (*M* = 3.26, *SD* = 0.47), though differences across cycles were not statistically significant (*p*=.071). Flow showed a very similar pattern: lower in Cycle 1 (*M* = 4.73, *SD* = 0.88) and higher in Cycles 2 (*M* = 5.42, *SD* = 0.91) and 3 (*M* = 5.27, *SD* = 0.68; *p*=.185). The consistent direction of the effect across both measures is consistent and suggests that the final, playable prototypes were successful in eliciting ratings of presence and flow that were comfortably above the mid-point of each item.

#### Engagement with the premise

In Cycle 1, qualitative data indicated that most participants engaged with the symbolic premise of the experience, but not all. Some participants thought that the items shown in images of the apartment scene represented literal objects, rather than tokens that represented important life priorities, and chose objects based on what they personally wanted (e.g., “*I love fabrics, so I’d keep the curtains”*). Those who did understand that the objects represented potential life priorities genuinely reflected on them: “*I would definitely say I'm future oriented and looking for a family. It’s definitely thought-provoking if you havent’ sat down and thought about values and priorities before*” (Participant #5, Cycle 1), and “*it’s that baggage of alcohol that’s like filling it all up. It was very real in that sense. I was like, yes, it is taking up space in my life. Look at it*” (Participant #1, Cycle 1). In Cycle 2, participants’ understanding of the premise improved substantially. By the truck scene, all participants understood the task, and many engaged with the metaphor spontaneously and meaningfully: “*I appreciate the metaphor of making room for what you actually value. That was a good little thought exercise, kind of in what your actual priorities are*” (Participant #13, Cycle 2), and “*I see the entire thing as a metaphor for life. Like, you're gonna have to think about these things in life. You cant’ ride through like it’s nothing. I like it. It was a very symbolic adventure*” (Participant #18, Cycle 2). In Cycle 3, premise engagement was nearly universal and qualitative responses often reflected spontaneous and deep reflection on the discrepancy between important life priorities and drinking: “*I think that when I've thought about wanting to like cut back, I dont’ think I've framed it in terms of like what is this taking away from like other parts of my life*” (Participant #30, Cycle 3), and “*to so explicitly present the idea that there's like a cost associated with spending your time drinking, it takes time away from doing other things, it just makes you think about that idea more*” (Participant #35, Cycle 3). These results are consistent with participants’ ratings of their actual-ideal discrepancy collected after the interviews, with ratings at or above the midpoint of the item in Cycles 2 and 3 (Cycle 2: *M* = 4.7, *SD* = 1.4, Cycle 3: *M* = 5.3, *SD* = 2.4), suggesting that many participants recognize some gap between their current drinking and their personal ideal.

#### Perceived MI-consistent tone

Participants' ratings of the perceived MI-consistent tone of the concept or prototypes were generally favorable across all three cycles (Supplemental Table 2). Participants strongly agreed that the session helped them reflect on what they wanted, prompted them to think about their life goals, and encouraged consideration of how their drinking aligned with their priorities (all cycles *M* ≥ 4.25 on a 1–5 scale). Participants disagreed that the session was judgmental, expressed disapproval, or told them what to do (all cycles M ≤ 2.38). Two items differed significantly across cycles: perceived empathy and support for independent decision-making, both of which were lower in Cycle 1 than in subsequent cycles. See Supplemental Table 2.

#### Most memorable and perspective-changing BMI topics

In Cycle 3, we assessed which of the topics covered in the mock BMIs stood out to participants, since this was the only cycle in which participants were exposed to all components of the planned BMI (Table 4). The VR experience was the most frequently selected topic that stood out the most (87.5%) and was most often ranked first (31.3%), giving it the highest rank score (4.38), followed by drinking patterns (3.25) and comparisons with other peers (2.63). When asked which topic changed their perspective the most, comparison with other students earned the highest rank score (3.56), having been picked by 12 participants (75.0%) and ranked first by 6 (37.5%). The VR experience was second (3.00), picked by 10 participants (62.5%) and ranked first by 2 (12.5%).

## Discussion

In this study, we developed *Fresh Start*, a VR experience intended to help counselors elicit change talk within brief motivational interventions (BMI), with the help of feedback from intended users across three iterative build-test-refine cycles. Across cycles, participants found the experience acceptable, feasible, and appropriate for use within a counseling session, with ratings at or near the top of each scale and a tendency toward higher ratings as prototype fidelity increased. *Fresh Start* also earned above average ratings for usability, and it was safe and well-tolerated, with no adverse events and generally mild symptoms. Most importantly, participants engaged with the core premise of the experience, and many spontaneously reflected on the gap between their drinking and the things they value most.

Our findings suggest that the *Fresh Start* experience, and using VR within alcohol-related counseling sessions generally, is acceptable among young adults. All participants agreed to try VR, even those with no experience with it, with several participants noting that the experience felt more engaging than a standard conversation. These findings are consistent with other usability studies, suggesting high interest in VR among various populations, including patients with substance use disorder in inpatient treatment (Arissen et al., 2022; Freeman et al., 2023; Garcia-Palacios et al., 2007; Levy et al., 2023). Participants also rated integrating VR into counseling sessions as feasible, with several describing it as fitting smoothly into the flow of a session, rather than interrupting it. This finding is key given that a tool that is engaging but hard to integrate would provide limited clinical value. It also adds to the now sizable body of evidence that VR can be successfully used within counseling sessions and in a variety of mental health-related clinical settings (Dellazizzo et al., 2020; Freeman et al., 2017).

Our results also suggest that the *Fresh Start* experience was usable. Nearly all participants in Cycles 2 and 3 were able to fit the headset within three minutes and orient to the controls and ultimately complete the experience without outside instruction, which was our goal. Participant feedback about usability problems throughout each cycle helped ensure that subsequent cycles improved upon any issues that were discovered. Ratings on the SUS were also above average, which is impressive for a relatively complex system like VR that most participants had little recent experience with.

The experience was also safe and well-tolerated. Symptoms were limited to mild or moderate dizziness, headache, and nausea, with minimal interference and low SSQ scores in every cycle. However, while the symptoms participants reported were largely mild, nearly half of participants in Cycle 3 reported at least one symptom after using VR. These symptoms were all consistent with mild cybersickness (e.g., nausea, dizziness, headache). Previous opinion has raised some concern about the potential for more severe adverse effects with VR, such as falls or injuries (Lundin et al., 2023). Our results do not support these concerns, particularly since our experience does not require users to move to navigate it. Still, the prevalence of mild symptoms is notable, given that even minor discomfort could be sufficient to reduce uptake or prompt early discontinuation. As a result, we made several changes to the final scene intended to increase comfort, including changes to how users navigate in this scene and steps to ensure that additional comfort settings are highlighted. Craving for a alcohol did not change within sessions in any cycle, suggesting that the alcohol depicted in the experience does not induce the urge to drink, a concern that is frequently raised about the safety of using VR experiences related to substance use (Ghiţă & Gutiérrez-Maldonado, 2018).

One of the most encouraging results of this study was that *Fresh Start* appeared to make the discrepancy between important life priorities and participants’ current drinking tangible and prompted them to reflect on it. By Cycle 3, nearly all participants engaged with the premise of the experience without prompting, often responding to general prompts about what they took away from the experience (“what did that make you think about?”) by framing their drinking in terms of what it takes away from other priorities. Ratings of MI-consistent tone were also strong, demonstrating that participants perceived the experience as non-judgmental and supportive of participants’ autonomy. That the experience is consistent with MI’s tone should further support ease of integrating a VR experience like this into MI-inspired counseling sessions. Finally, participants’ ratings of which counseling topics were most memorable and perspective-shifting were also encouraging. In general, the VR experience competed with normative comparisons as one of the most notable parts of the session and one of the most important in shifting their perspective on their drinking. Given the decades of research focused on the importance and effects of normative feedback in brief interventions (Lewis & Neighbors, 2006; M. B. Miller et al., 2013), we believe these results suggest that VR has the potential to become a useful component of BMIs. Together, these results suggest that *Fresh Start* is ready for more rigorous testing to evaluate its impact on meaningful patient outcomes.

Several limitations are important to note. First, although there is robust evidence that as few as five participants are sufficient for finding almost all usability problems with software systems (Nielsen & Landauer, 1993; Virzi, 1992), such small samples are less likely to produce robust results about other questions, such as whether the experience reliably shifted participants’ perspectives about their drinking. Future research will address this question. Our samples were also largely White, female, and full-time students, which limits generalizability. Next, nearly all measures collected were self-report, and although most participants completed the post-intervention survey on their own at home within a few days of their session, these responses could still be subject to demand characteristics, particularly since participants were aware this experience was under development.

In summary, *Fresh Start* is an acceptable, feasible, and safe way to incorporate VR into brief motivational interventions for young adults, and it appears to reliably prompt the sort of self-reflection on drinking patterns that it was designed to elicit. This work addresses an important gap in the literature that has used VR almost exclusively for cue exposure and demonstrates that VR has potential for promoting change in other ways. Our approach of grounding the design in iterative feedback cycles from users also provides more confidence that it reflects the perspectives of its intended users. Our next step is to conduct a fully-powered, randomized controlled trial to test whether integrating *Fresh Start* into a BMI helps young adults reduce their alcohol use and related problems relative to BMI alone. If it is effective, this work will help establish VR as a tool for motivating behavior change and create a new pathway for strengthening MI-inspired interventions.

## Supplementary Material

Supplementary Files

This is a list of supplementary files associated with this preprint. Click to download.

- SupplementalTables.docx

## Figures and Tables

**Figure 1:**
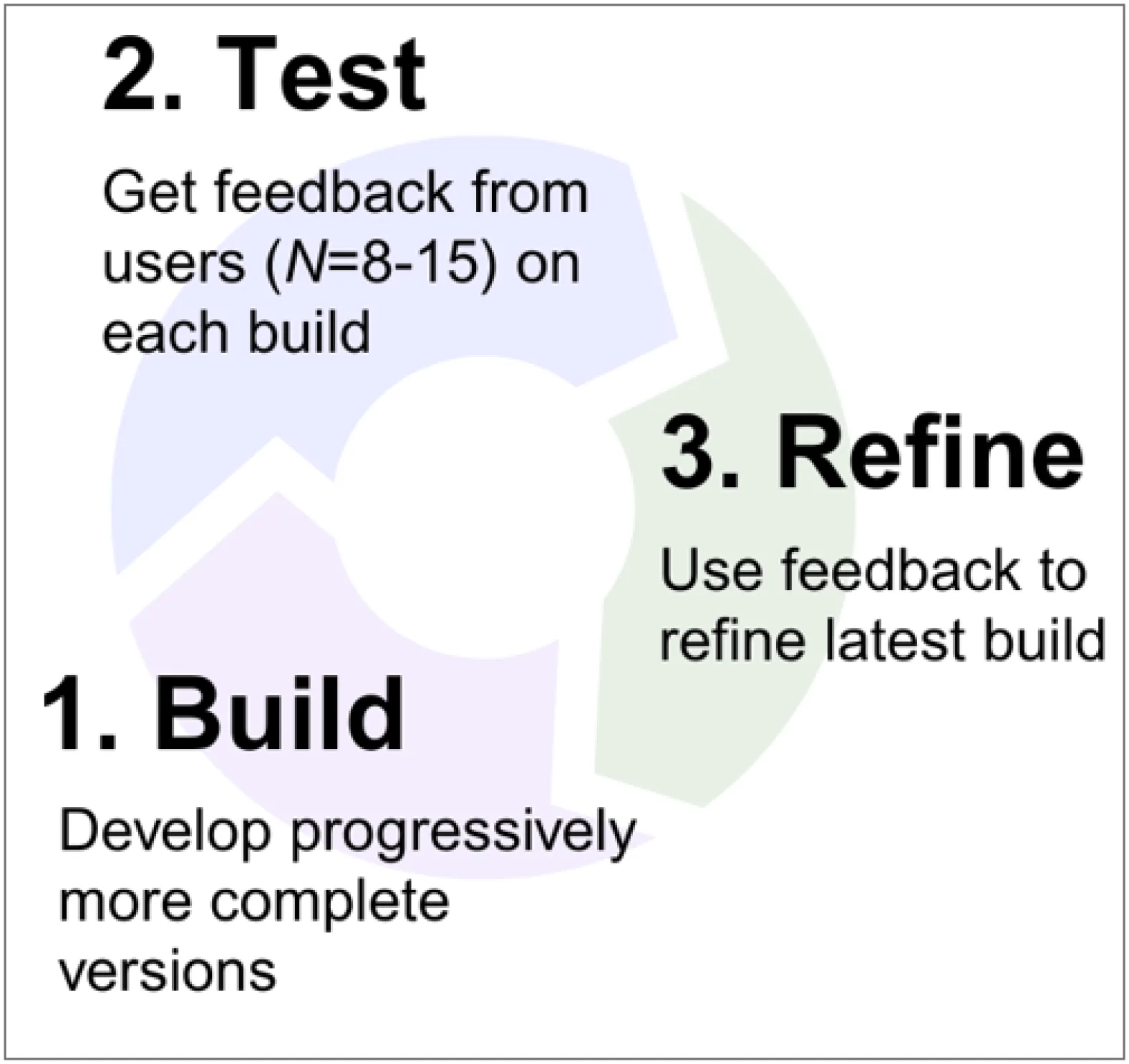
Build-Test-Refine cycles of user research used to develop the *Fresh Start* VR experience

**Figure 2:**
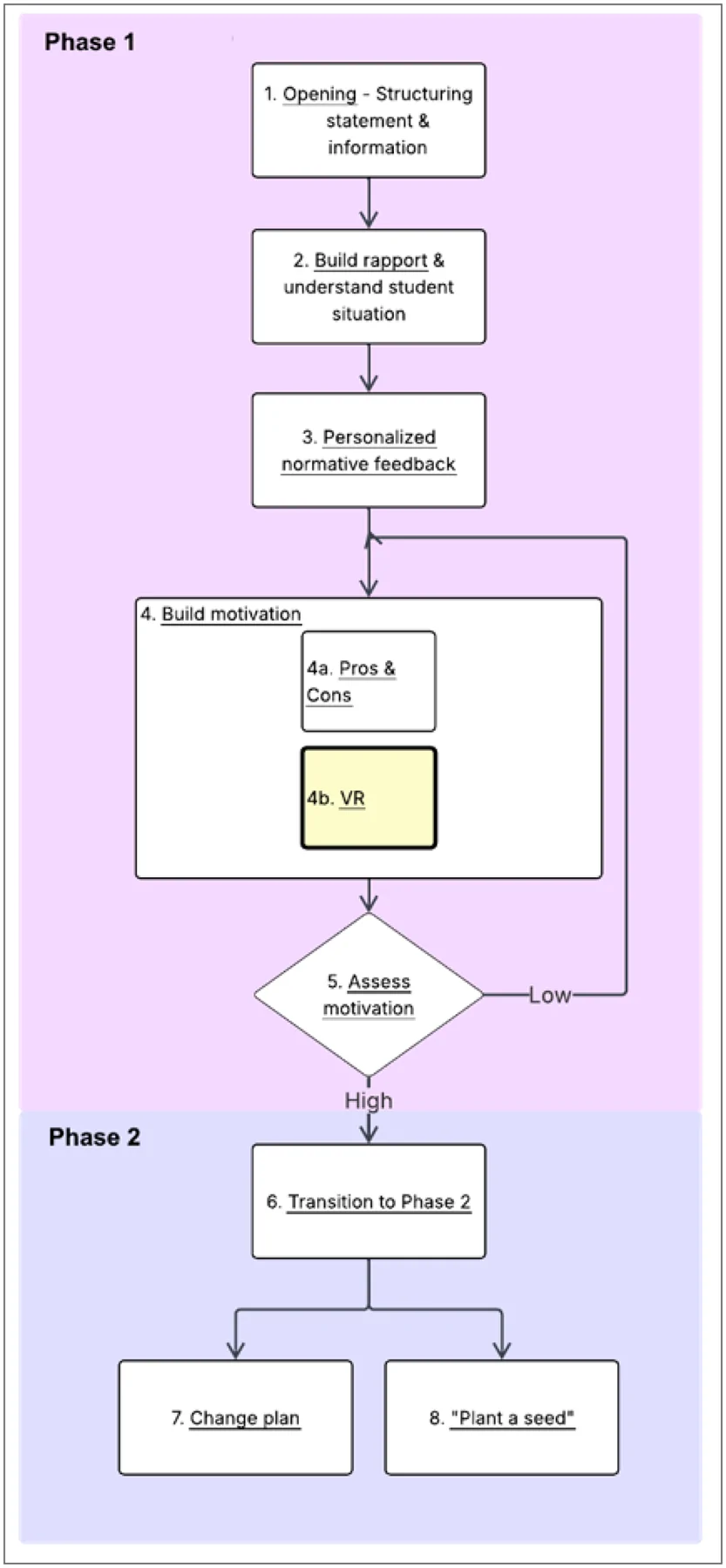
Flow of BMI sessions and topics

**Figure 3:**
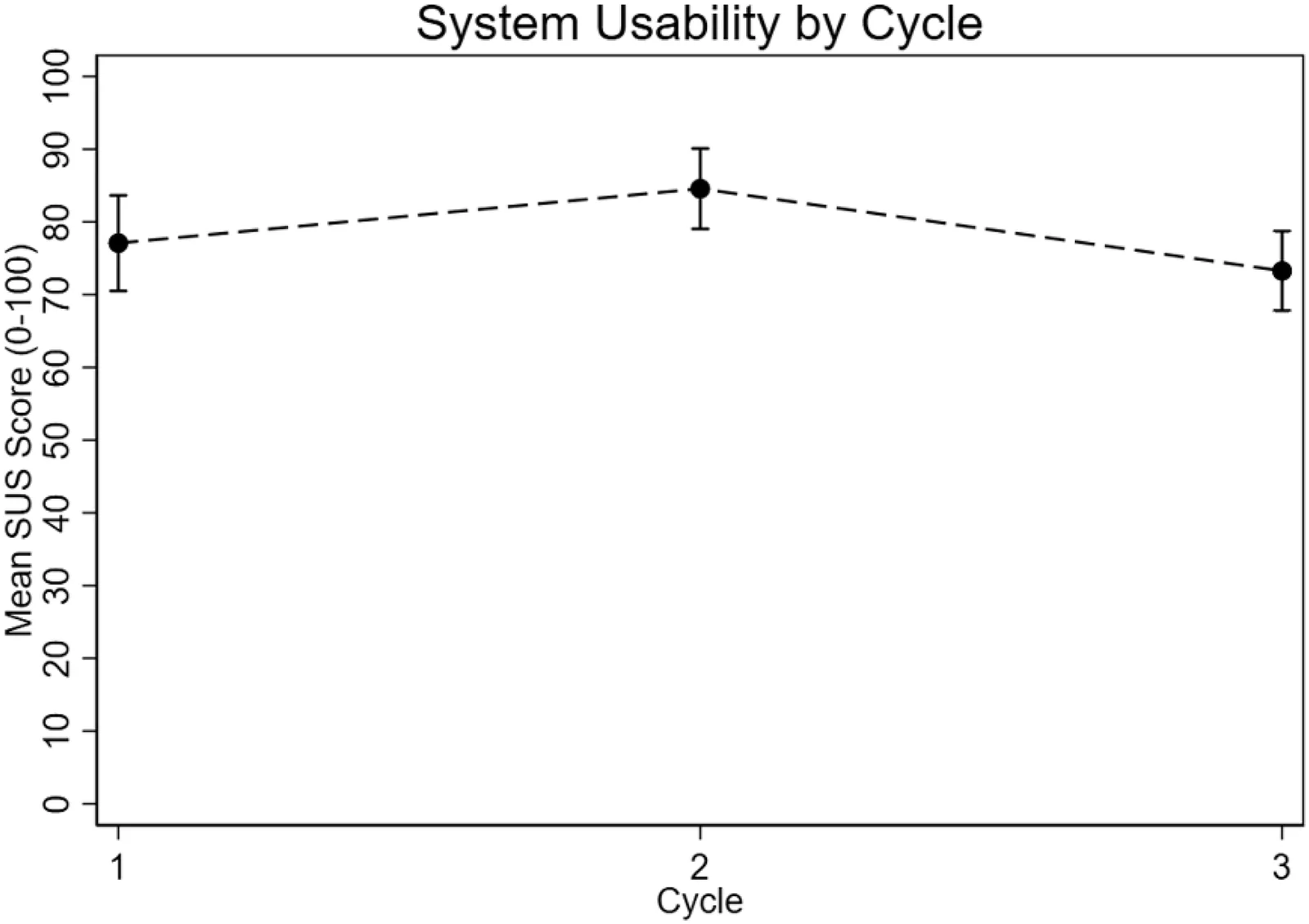
System Usability Scale total scores by cycle

**Figure 4:**
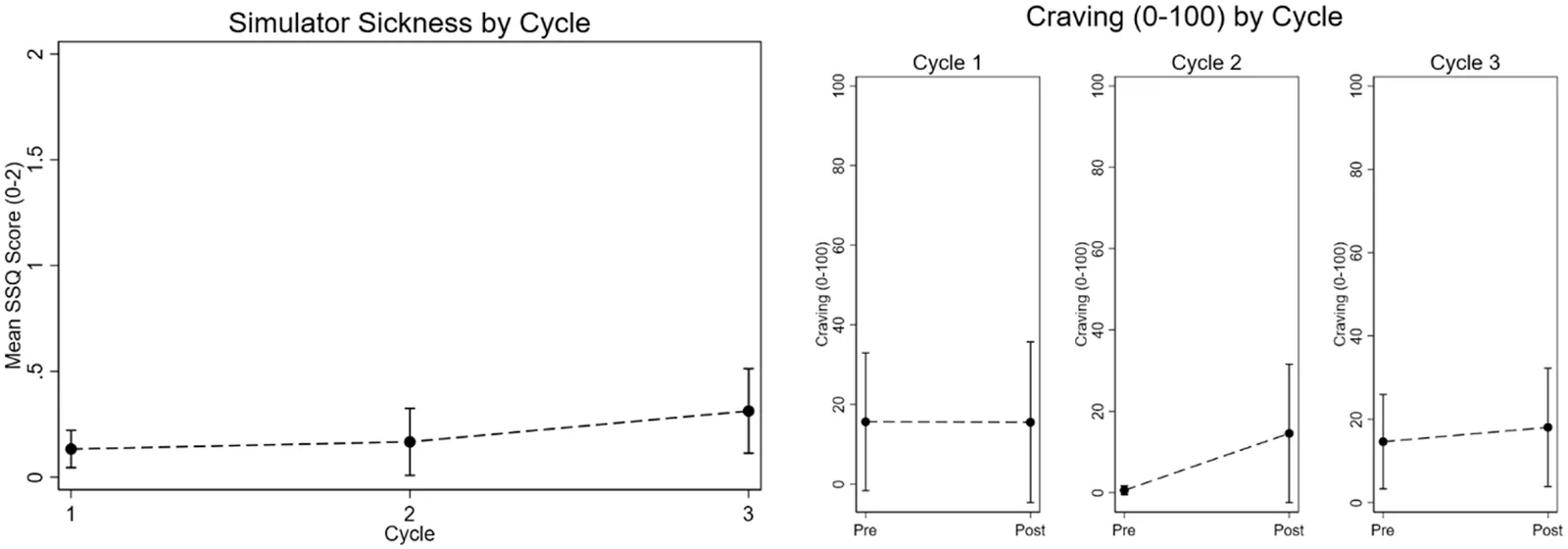
Simulator Sickness Questionnaire (SSQ) total scores (left panel) and alcohol craving visual analogue scale ratings (right panel) by cycle

**Figure 5:**
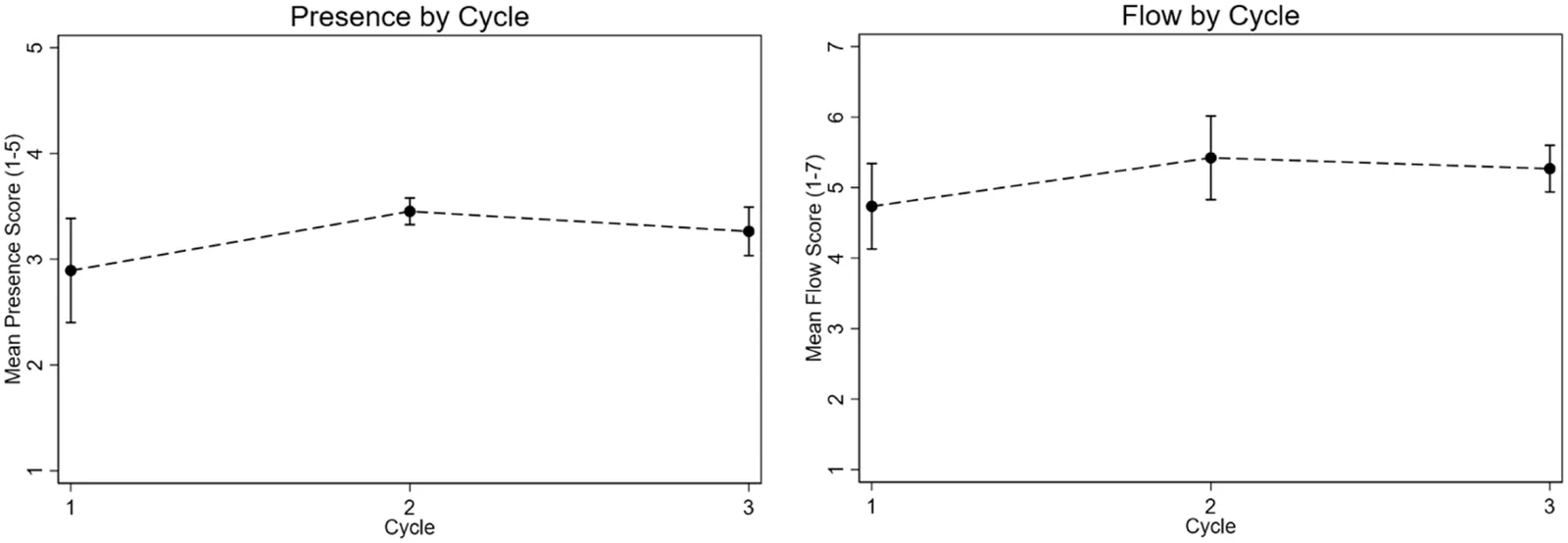
Participants’ ratings of subjective presence and flow while immersed in VR by cycle

**TABLE 1.** Participant demographic and baseline characteristics (*N*=32)

Characteristics	Overall (N=32)	Cycle 1 (N=8)	Cycle 2 (N=9)	Cycle 3 (N=15)
Age (years)	22.8 (3.3)	21.4 (2.5)	22.3 (2.7)	23.9 (3.7)
**Gender**				
woman	19 (57.6)	4 (50.0)	4 (44.4)	11 (68.8)
man	14 (42.4)	4 (50.0)	5 (55.6)	5 (31.3)
**Sex assigned at birth**				
female	19 (57.6)	4 (50.0)	4 (44.4)	11 (68.8)
male	14 (42.4)	4 (50.0)	5 (55.6)	5 (31.3)
Ethnicity (Hispanic or Latino)				
not Hispanic	27 (81.8)	7 (87.5)	7 (77.8)	13 (81.3)
Hispanic	6 (18.2)	1 (12.5)	2 (22.2)	3 (18.8)
**Race**				
White	26 (81.3)	5 (71.4)	8 (88.9)	13 (81.3)
Black	2 (6.3)	0 (0.0)	1 (11.1)	1 (6.3)
Asian	4 (12.5)	2 (28.6)	0 (0.0)	2 (12.5)
**Relationship status**				
single	19 (57.6)	4 (50.0)	5 (55.6)	10 (62.5)
committed relationship	10 (30.3)	2 (25.0)	3 (33.3)	5 (31.3)
domestic partnership	3 (9.1)	2 (25.0)	0 (0.0)	1 (6.3)
married	0 (0.0)	0 (0.0)	0 (0.0)	0 (0.0)
other	1 (3.0)	0 (0.0)	1 (11.1)	0 (0.0)
**Education**				
GED	1 (3.0)	0 (0.0)	0 (0.0)	1 (6.3)
some college	13 (39.4)	4 (50.0)	4 (44.4)	5 (31.3)
bachelors degree	9 (27.3)	2 (25.0)	2 (22.2)	5 (31.3)
some grad school	2 (6.1)	0 (0.0)	1 (11.1)	1 (6.3)
master's degree	2 (6.1)	0 (0.0)	1 (11.1)	1 (6.3)
some doctoral school	5 (15.2)	2 (25.0)	1 (11.1)	2 (12.5)
**Annual household income**				
$0–29k	19 (59.4)	4 (50.0)	5 (55.6)	10 (66.7)
30k-59k	7 (21.9)	3 (37.5)	1 (11.1)	2 (13.3)
60–89k	6 (18.8)	1 (12.5)	3 (33.3)	3 (20.0)
**Employment status**				
unemployed	1 (3.0)	0 (0.0)	0 (0.0)	1 (6.3)
part-time	5 (15.2)	2 (25.0)	1 (11.1)	2 (12.5)
full-time	11 (33.3)	2 (25.0)	4 (44.4)	5 (31.3)
full-time student	16 (48.5)	4 (50.0)	4 (44.4)	8 (50.0)
**High (>16) AUDIT score**	4 (12.1)	2 (25.0)	0 (0.0)	2 (12.5)
**Ever used VR**				
No	9 (27.3)	2 (25.0)	2 (22.2)	5 (31.3)
Yes	24 (72.7)	6 (75.0)	7 (77.8)	11 (68.8)
**VR use frequency**				
not in the past year	10 (41.7)	2 (33.3)	1 (14.3)	7 (63.6)
once or twice in past year	13 (54.2)	3 (50.0)	6 (85.7)	4 (36.4)
several times in past year, but not in past month	1 (4.2)	1 (16.7)	0 (0.0)	0 (0.0)
at least once in past month	0 (0.0)	0 (0.0)	0 (0.0)	0 (0.0)

*Note*. Percentages based on non-missing values.

**TABLE 2.** Intervention measure scores by cycle

Measure	Cycle 1 (*N*=8) *M (SD)*	Cycle 2 (*N*=9) *M (SD)*	Cycle 3 (*N*=15) *M (SD)*	ANOVA *p*
Acceptability of Intervention Measure (AIM)	3.97 (0.85)	4.50 (0.47)	4.27 (0.44)	0.176
Feasibility of Intervention Measure (FIM)	4.59 (0.38)	4.50 (0.56)	4.20 (0.43)	0.110
Appropriateness of Intervention Measure (APIM)	3.66 (1.18)	4.33 (0.54)	4.22 (0.44)	0.119
System Usability Scale (SUS)	77.08 (9.48)	84.57 (8.46)	75.00 (9.03)	0.040

*Note*. p-value from one-way ANOVA comparing cycles. AIM, FIM, and APIM totals represent average item rating on a 1–5 scale. SUS scored 0–100.

**TABLE 3.** Presence and flow scores by cycle

Measure	Cycle 1 (N=8) *M (SD)*	Cycle 2 (N=9) *M (SD)*	Cycle 3 (N=15) *M (SD)*	*F*	*p*
Presence (1–5)	2.89 (0.71)	3.45 (0.19)	3.26 (0.47)	2.885	0.071
Flow (1–7)	4.73 (0.88)	5.42 (0.91)	5.27 (0.68)	1.788	0.185
Simulator Sickness Questionnaire (SSQ) (0–2)	0.13 (0.13)	0.17 (0.24)	0.31 (0.41)	1.076	0.354

*Note*. p-value from one-way ANOVA comparing cycles. Means represent average item ratings.

**TABLE 4.** Most salient counseling topics among Cycle 3 participants (pick-and-rank)

Counseling topic	Picked N (%)	Ranked #1 N (%)	Mean rank (SD)	Median rank	Rank score
**Most memorable**					
Typical drinking patterns	12 (75.0)	2 (12.5)	2.67 (1.50)	2.0	3.25
Comparison with other students	10 (62.5)	4 (25.0)	2.80 (1.75)	3.0	2.63
Pros and cons of drinking	9 (56.3)	2 (12.5)	2.56 (1.13)	3.0	2.50
Priorities & how drinking fits (VR)	14 (87.5)	5 (31.3)	2.00 (0.96)	2.0	4.38
Interest & confidence to change	6 (37.5)	0 (0.0)	5.33 (0.52)	5.0	0.63
How/when to change (change plan)	11 (68.8)	1 (6.3)	3.45 (1.51)	3.0	2.44
					
**Changed perspective the most**					
Typical drinking patterns	8 (50.0)	2 (12.5)	3.50 (2.27)	3.0	1.75
Comparison with other students	12 (75.0)	6 (37.5)	2.25 (1.60)	1.5	3.56
Pros and cons of drinking	8 (50.0)	2 (12.5)	2.88 (1.64)	2.5	2.06
Priorities & how drinking fits (VR)	10 (62.5)	2 (12.5)	2.20 (1.14)	2.0	3.00
Interest & confidence to change	7 (43.8)	1 (6.3)	3.29 (1.80)	3.0	1.63
How/when to change (change plan)	9 (56.3)	1 (6.3)	3.11 (1.17)	3.0	2.19

*Note*. Percentages use all Cycle 3 participants as the denominator. Mean and median rank are computed among participants who picked each topic.

## Data Availability

The datasets generated and/or analyzed during the current study will be made available after study completion. A de-identified archival dataset will be uploaded to the NIMH Data Archive and made available to requesting investigators after an embargo period of 2 years.
